# P-1016. A Deeper Look at an Uncommon Opportunist: *Geotrichum capitatum* Bloodstream Infections at a Cancer Center

**DOI:** 10.1093/ofid/ofae631.1206

**Published:** 2025-01-29

**Authors:** Sung-Yeon Cho, Sebastian Wurster, Takahiro Matsuo, Nathaniel D Albert, Jeffery Tarrand, Dimitrios P Kontoyiannis

**Affiliations:** Division of Infectious Diseases, Department of Internal Medicine, College of Medicine, The Catholic University of Korea, Seoul, Korea, Seoul, Seoul-t'ukpyolsi, Republic of Korea; The University of Texas MD Anderson Cancer Center, Houston, Texas; The University of Texas MD Anderson Cancer Center, Houston, Texas; The University of Texas MD Anderson Cancer Center, Houston, Texas; University of Texas MD Anderson Cancer Center, Houston, TX, Houston, Texas; The University of Texas MD Anderson Cancer Center, Houston, Texas

## Abstract

**Background:**

Opportunistic non-*Candida* yeasts such as *Geotrichum capitatum* have been increasingly encountered in cancer patients (pts). Clinical and microbiological characteristics of this uncommon pathogen have not been studied.

Comparison of virulence between Geotrichum capitatum and Candida albicans using survival analysis in Toll-deficient fruit flies (Drosophila melanogaster) infection model (Mantel Cox Log-rank test)

**Figure:**
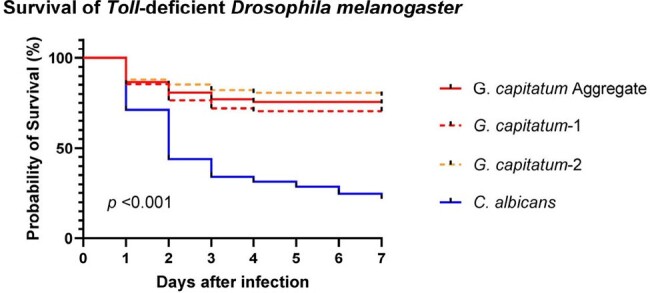

**Methods:**

We retrospectively reviewed all *G. capitatum* bloodstream infections (BSIs) in adult pts with hematologic malignancies at MD Anderson Cancer Center (2013–2022). We also compared *in-vitro* biofilm production and virulence of two available *G. capitatum* bloodstream isolates versus *Candida albicans* (control) in a *Toll*-deficient *(Tl-)* fruit fly infection model.

**Results:**

Fifteen pts had *G. capitatum* BSIs. All pts were neutropenic (neutrophil count < 500/µL) and had an indwelling catheter but only one episode was deemed to be catheter-associated. All BSIs were breakthrough infections to prior antifungals, mostly caspofungin-based regimens (n=8, 53%). Incidence rate was 0.095/1,000 pt-days in 2022, with an average 1.58-fold annual increase since 2013 (*p*=0.001). Most pts (n=13, 87%) presented with septic shock/sepsis at the onset of BSI and 73% (n=11) developed multiorgan failure during the course. Gastrointestinal mucosal barrier injury (MBI; n=11, 73%) and dissemination (n=13, 87%) (i.e., septic emboli, hepatosplenic involvement) were common. Forty-two-day mortality was 60%. Neutrophil recovery was observed in all 6 survivors but only in 44% (4/9) of non-survivors (*p*=0.086). Minimum inhibitory concentrations were 2–8, 0.03–0.25, 0.125–1, 4, 0.5–4, and 0.5–2 µg/mL for fluconazole, voriconazole, posaconazole, caspofungin, anidulafungin, and amphotericin B, respectively. In 6 of 8 pts tested, serum β-D-Glucan was >80 pg/mL (median 352, range 95–500). *G. capitatum* infection of *Tl-* flies resulted in lower 7-day mortality than *C. albicans* infection (22 *vs*. 74%, *p*< 0.001) (Fig). *G. capitatum* strains produced biofilms *in vitro*, albeit less than *C. albicans* at the same inoculum. There was inter-strain variability in both virulence and biofilm production.

**Conclusion:**

*G. capitatum* BSIs are increasing and are commonly associated with breakthrough nature, neutropenia, and MBI. Lower virulence compared to *C. albicans* in the fly model and the correlation of pts survival with neutrophil recovery point to host-driven factors as important determinants of outcome.

**Disclosures:**

**Sebastian Wurster, MD, MSc**, Astellas Pharma: Grant/Research Support|Gilead Sciences: Grant/Research Support **Dimitrios P. Kontoyiannis, MD**, AbbVie: Advisor/Consultant|Astellas Pharma: Advisor/Consultant|Astellas Pharma: Grant/Research Support|Astellas Pharma: Honoraria|Cidara Therapeutics: Advisor/Consultant|Gilead Sciences: Advisor/Consultant|Gilead Sciences: Grant/Research Support|Gilead Sciences: Honoraria|Knight: Advisor/Consultant|Merck: Advisor/Consultant|Scynexis: Advisor/Consultant

